# De-PEN-SAFE: Delabeling der anamnestischen „Penicillinallergie“ mittels oraler Exposition gegen Amoxicillin: Ein multizentrisches prospektives Forschungsprojekt zur Evaluation von Allergien gegen β-Lactam-Antibiotika

**DOI:** 10.1007/s00101-025-01571-0

**Published:** 2025-07-23

**Authors:** Musa Ayoub, Marcus Bauer, Reinhard Bornemann, Andreas Heidenreich, Maximilian David Mauritz, Annika Hoyer, Janina Soler Wenglein, Anke Hildebrandt

**Affiliations:** 1 Medizinische Fakultät, Universität Münster, Münster, Deutschland; 2 Klinik für Anästhesiologie, St. Vincenz-Krankenhaus Datteln, Rottstraße 11, 45711 Datteln, Deutschland; 3 Medizinische Klinik II, St. Vincenz-Krankenhaus Datteln, Datteln, Deutschland; 4 Innere Klinik, Klinikum Bielefeld, Bielefeld, Deutschland; 5 Arbeitsgruppe 2 Bevölkerungsmedizin und biomedizinische Grundlagen, Fakultät für Gesundheitswissenschaften, Universität Bielefeld, Bielefeld, Deutschland; 6 Universitätsklinik für Kinder und Jugendmedizin, Evangelisches Klinikum Bethel, Medizinische Fakultät und Universitätsklinik OWL, Universität Bielefeld, Bielefeld, Deutschland; 7 Institut für Sozialmedizin und Epidemiologie, Universität zu Lübeck, Lübeck, Deutschland; 8 Vestische Kinder- und Jugendklinik Datteln, Datteln, Deutschland; 9 Lehrstuhl für Pädiatrie, Department für Humanmedizin, Medizinische Fakultät, Universität Witten/Herdecke, Witten, Deutschland; 10 Biostatistik und Medizinische Biometrie, Medizinische Fakultät OWL, Universität Bielefeld, Bielefeld, Deutschland; 11 Institut für Medizinische Mikrobiologie, Universitätsklinikum Münster, Münster, Deutschland; 12 Medizinische Klinik I, St. Vincenz-Krankenhaus Datteln, 45711 Datteln, Deutschland

## Hintergrund & Hypothesen

Die anamnestische Angabe einer „Penicillinallergie“ ist ein signifikantes Problem im Gesundheitswesen. Etwa 5–15 % der Menschen in Ländern mit hohem Einkommen geben anamnestisch eine „Penicillinallergie“ an und tragen somit das Label „Penicillinallergie“ in ihrer Gesundheitsakte [1, 2]. Mehr als 90 % der Menschen mit anamnestischer „Penicillinallergie“ zeigen jedoch keine entsprechende Reaktion in allergologischen Tests oder bei erneuten Penicillinexpositionen im Rahmen einer Provokationstestung [3].

Viele Kliniken und Arztpraxen reagieren auf eine entsprechende Anamnese mit dem Vermeiden der Penicilline. Die Fehldiagnose einer „Penicillinallergie“ stellt daher ein bedeutendes klinisches Problem dar, da sie in der Konsequenz mit einer vermehrten Verordnung von Breitspektrumantibiotika, längeren Krankenhausaufenthalten und einer erhöhten Rate an Resistenzentwicklungen assoziiert ist [4].

In Deutschland gibt es dazu zwei S2k-Leitlinien: aus dem Jahr 2019 für β‑Lactam-Hypersensitivitätsreaktion und aus dem Jahr 2023 für medikamentöse Hypersensitivitätsreaktion [5, 6]. Hier ist an erster Stelle eine allergologische Testung mittels Hauttests empfohlen, die orale Provokationstestung (OPT) spielt hingegen nur eine untergeordnete Rolle. Ein aktuelles Positionspapier für den deutschsprachigen Raum (Deutschland, Österreich, Schweiz) schlägt ein differenzierteres Vorgehen vor, das eine orale Reexposition nach entsprechender Risikostratifizierung beinhaltet [7]. Die Durchführung eines allergologischen Hauttests in der Praxis ist teuer, zeitaufwendig und in der Breite nur gering verfügbar [5, 8, 9]. Ein Update der S2k-Leitlinie für Erwachsene und eine Leitlinie der Europäischen Gesellschaft für Klinische Mikrobiologie und Infektionskrankheiten (ESCMID) zum Vorgehen bei einer anamnestischen „Penicillinallergie“ sollen in Kürze erscheinen. Im klinischen Alltag existieren viele Herausforderungen für ein einheitliches Vorgehen, u. a. bei der Auswahl der Testung, deren Durchführung und anschließendem Delabeling einer sich als unzutreffend herausstellenden „Penicillinallergie“.

Wir möchten daher im Rahmen des ABS-Netzwerkes Westfalen-Lippe mittels einer Diagnosegütestudie Daten an zwei Standorten der Ärztekammer Westfalen-Lippe jeweils in der Erwachsenenmedizin und der Pädiatrie erheben. Ziele sind, das Vorliegen einer tatsächlichen „Penicillinallergie“ bei hospitalisierten Personen der Allgemeinbevölkerung zu überprüfen und ein standardisiertes Vorgehen im Rahmen eines Delabeling zu etablieren. In dieser Veröffentlichung stellen wir das Vorgehen des Delabeling in der Erwachsenenkohorte vor.

Der innovative Ansatz unserer Studie besteht darin, keine hausinterne Risikostratifizierung zu entwickeln, sondern vielmehr den bereits in der Literatur bewährten PEN-FAST-Score als Instrument zur Einschätzung des Risikos zu verwenden [9]. Wir wollen damit ein klinisches Vorgehen, welches sich ggf. auch auf andere Häuser leicht übertragen lässt, etablieren. Ein weiteres Ziel im Erwachsenenbereich ist das Schaffen einer Evidenz zu Sicherheit und Effektivität der praxisnahen Therapiedosis als Testdosis.

## Details der Studie

Unsere Studie ist multizentrisch, altersübergreifend und prospektiv. In Datteln und Bielefeld untersuchen wir Patient:innen systematisch nach einem standardisierten Vorgehen zur oralen Provokationstestung (OPT) und führen, wenn möglich, ein Delabeling durch.

### Studiendesign

**Abb. 1:**
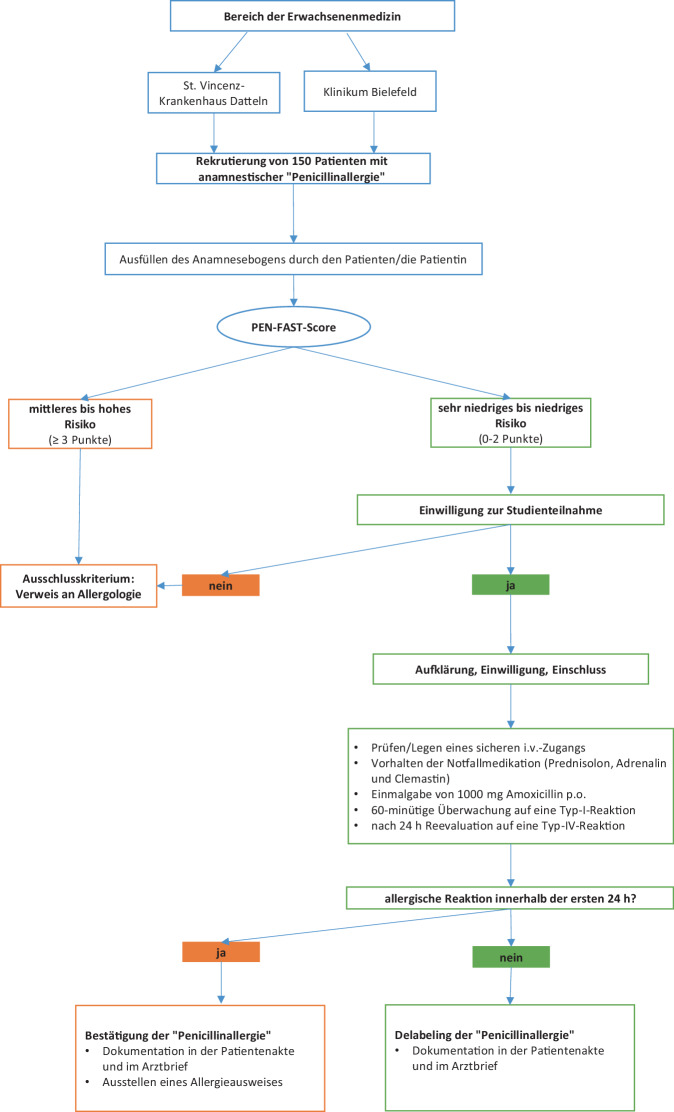
*Vorgehen im Studienarm der Erwachsenen*. Wir planen den Einschluss von 150 Patient:innen nach vorheriger Risikostratifizierung entsprechend dem PEN-FAST-Score. Bleiben in der oralen Provokationstestung in den ersten 24 h nach Einnahme einer Einmaldosis von Amoxicillin 1000 mg p.o. allergische Symptome aus, ist ein Delabeling möglich. Andernfalls erhalten die Patient:innen einen Allergieausweis. Im Bereich der Pädiatrie arbeiten die beteiligten Studienzentren mit einem anderen Score zur Risikostratifizierung und einem separaten Studienprotokoll.

Teilnehmen können Patient:innen, die sich gerade in stationärer Behandlung befinden und bei denen eine „Penicillinallergie“ in der Anamnese berichtet oder in den Unterlagen ohne weitere Erläuterung der Symptomatik dokumentiert ist. Das Vorgehen in der Erwachsenenmedizin ist in Abb. 1 dargestellt. Für pädiatrische Patient:innen existiert ein separates Studienprotokoll.

### Datenerhebung und Qualitätsbewertung

Datensammlung und -auswertung finden fortlaufend statt, sobald Patient:innen in die Studie eingeschlossen sind. Die Dokumentation umfasst Anamnesebogen, PEN-FAST-Bogen, Einwilligungen, Checklisten, Überwachungsbogen, Abschlussdokumentationen, Delabeling-Bescheinigung oder Bestätigung einer Allergie mit Ausstellen eines Allergiepasses. Nach Anonymisierung der Daten ist eine wissenschaftliche Auswertung geplant.

### Endpunkte

Primärer Endpunkt ist der positive Nachweis einer anamnestisch berichteten „Penicillinallergie“ durch eine Ja/Nein-Antwort. Zu sekundären Endpunkten zählen Anzahl und Art unerwünschter Arzneimittelreaktionen (UAW), Dokumentation einer Typ-I- und Typ-IV-Reaktion, Probleme bei der praktischen Umsetzung der direkten oralen Provokation, Compliance bei der Nachbeobachtung sowie anamnestische Angaben, die mit der Annahme einer Allergie assoziiert sind.

## Statistik

### Eigene Pilotdaten

Im St. Vincenz-Krankenhaus in Datteln erhalten Erwachsene mit anamnestischer „Penicillinallergie“ im Rahmen der täglichen Patientenversorgung und der ABS-Konsile – unabhängig von der aktuellen Studie – bereits seit ca. 2 Jahren eine OPT-Testung mittels Amoxicillin. Bislang traten keine Typ-I-Reaktion und eine Typ-IV-Reaktion vom Spättyp in 2 von insgesamt 80 (2,5 %) Patient:innen auf.

### Fallzahlplanung

Die Fallzahlplanung für die geplante Diagnosegütestudie erfolgte für die co-primären Endpunkte Sensitivität und Spezifität nach dem Ansatz von Obuchowski [10] und zusätzlicher Berücksichtigung evtl. Studienabbrüche. Insgesamt streben wir eine Fallzahl von 150 Personen im Erwachsenenbereich an.

## Ethik

Zur Studie liegt ein positives Votum seitens der Ethikkommission der Ärztekammer Westfalen-Lippe vor (2024-837-f-S). Zudem ist die Studie beim Deutschen Register für Klinische Studien registriert (DRKS00035664).

## Meilensteine

Die Erhebungsdauer der Studie beträgt 2 Jahre ab Studienbeginn 01.06.2025 bzw. endet mit dem Erreichen der geplanten Studienteilnehmerzahl.

### Sicherheitsberichterstattung

Zum Zeitpunkt des Studieneinschlusses klären wir alle Patient:innen darüber auf, dass es zum Auftreten von UAW kommen kann. Jeglicher vermutete kausale Zusammenhang zwischen auftretender UAW und der jeweiligen Antibiotikaeinnahme ist dem Studienleiter:in des jeweiligen Studienstandorts zu melden.

Eine UAW definieren wir als individuelle schädliche und unerwartete Körperreaktion, die im Rahmen der prophylaktischen oder therapeutischen Antibiotikadosis auftritt. Um diese Reaktionen zu verhindern, haben wir Ausschlusskriterien definiert. Zudem findet vorab eine detaillierte Risikostratifizierung der Patient:innen statt.

### Individuelle Abbruchkriterien

Medizinische Abbruchkriterien: Bei erwachsenen Patient:innen erfolgt die Studie im Kern mit der Einmalgabe von Amoxicillin. Eine adverse Reaktion wäre eines der – wenngleich in geringem Ausmaß – erwarteten Outcomes als Bestätigung einer anamnestisch berichteten „Penicillinallergie“. Eine weitere Exposition bzw. Intervention ist darüber hinaus mit und ohne adverse Reaktion nicht vorgesehen, sodass medizinische Abbruchkriterien nicht anwendbar sind.

Zurückziehen der Einwilligung: Patient:innen können jederzeit ihre zuvor erteilte Einwilligung zur Teilnahme an der Studie zurückziehen. Hieraus ergibt sich kein Nachteile in Betreuung, Diagnostik und Therapie.

### Abbruchkriterien für die Studie insgesamt

Es erfolgt zunächst die Zielrekrutierung von 50 Patient:innen innerhalb der ersten 6 Monate Studienlaufzeit. Während dieser Laufzeit und auch im Weiteren erhält die Gesamtprojektkoordination zu jeder UAW aus jedem Zentrum eine anonymisierte Rückmeldung mit Kategorisierung der UAW (neben allergischen Reaktionen Dokumentation anderer unspezifischer Symptome wie z. B. Kopfschmerzen, Schwindel, Palpitationen, isolierte gastrointestinale Reaktionen). Übersteigt das Ausmaß der studienbedingt möglichen allergischen Reaktionen auf die Exposition mit einem Penicillinpräparat den Erwartungswert von mehr als standortübergreifend 2 % anaphylaktischer bzw. Typ-I-Reaktionen und mehr als 10 % Spättyp bzw. Typ-IV-Reaktionen, brechen wir die Studie nach dem ursprünglich vorgelegten Protokoll ab.

## Studiengruppe/Expertise

- *Gesamtprojektkoordination und Principal Investigator Standort Datteln Medizinische Klinik: *PD Dr. med. Anke Hildebrandt*, *Fachärztin für Medizinische Mikrobiologie, Virologie und Infektionsepidemiologie, Fachärztin für Innere Medizin und Infektiologie, Lehrbeauftragte der Universität Münster, Medizinische Fakultät, Institut für Medizinische Mikrobiologie, Domagkstr. 10, 48149 Münster, anke.hildebrandt@uni-muenster.de, Leitende Oberärztin Medizinische Klinik I, St. Vincenz-Krankenhaus Datteln, Rottstraße 11, 45711 Datteln, Tel.: +49 2363 108 2045, a.hildebrandt@vincenz-datteln.de*, *
- *Principial Investigator Campus Klinikum Bielefeld: *Prof. Dr. med. Dr. Public Health Reinhard Bornemann*, *Facharzt für Innere Medizin, Infektiologie, Epidemiologe DGEpi*, *Klinikum Bielefeld, Oberarzt Innere Medizin, Teutoburger Str. 50, 33604 Bielefeld, Tel. +49 521 581‑0, Universität Bielefeld, Fakultät für Gesundheitswissenschaften, AG 2 Bevölkerungsmedizin und Versorgungsforschung, Universitätsstr. 25, 33615 Bielefeld, Tel.: +49 521 106-6889, bornemann@uni-bielefeld.de
- *Weitere an der Studie beteiligte Personen: *Dr. med. Lutz Uflacker, Ärztlicher Direktor Gesamtklinikum und Chefarzt der Medizinischen Klinik I, St. Vincenz-Krankenhaus Datteln, Rottstraße 11, 45711 Datteln*; *Musa Ayoub, Oberarzt der Klinik für Anästhesie, St. Vincenz-Krankenhaus Datteln, Rottstraße 11, 45711 Datteln*; *Prof. Dr. med. Barbara Kahl, Oberärztin im Institut für Medizinische Mikrobiologie des Universitätsklinikums Münster, Domagkstraße 10, 48149 Münster
- *Biometriker: *Dr. rer. hum. biol. Andreas Heidenreich, M. A., Universitätsklinik für Kinder- und Jugendmedizin, Evangelisches Klinikum Bethel, Grenzweg 10, 33617 Bielefeld
- *Biometrische Beratung: *Prof. Dr. Annika Hoyer, Biostatistik und Medizinische Biometrie, Medizinische Fakultät OWL, Universität Bielefeld, Universitätsstraße 25, 33615 Bielefeld

## Sponsor/Finanzierung

- Geldgeber*in: Es existiert keine externe Finanzierung. Die Kosten der Studie tragen die beteiligten Studienzentren selbst.
- *Sachmittel: *Personal- und Sachmittel bringen die einzelnen Studienorte entsprechend des Studiendesigns selbst mit ein.

## References

[1] Lee CE, Zembower TR, Fotis MA et al (2000) The incidence of antimicrobial allergies in hospitalized patients: implications regarding prescribing patterns and emerging bacterial resistance. Arch Intern Med 160:2819. 10.1001/archinte.160.18.2819 11025792 10.1001/archinte.160.18.2819

[2] MacFadden DR, LaDelfa A, Leen J et al (2016) Impact of reported beta-lactam allergy on inpatient outcomes: a multicenter prospective cohort study. Clin Infect Dis 63:904–910. 10.1093/cid/ciw462 27402820 10.1093/cid/ciw462

[3] Chua KYL, Vogrin S, Bury S et al (2021) The penicillin allergy delabeling program: a multicenter whole-of-hospital health services intervention and comparative effectiveness study. Clin Infect Dis Off Publ Infect Dis Soc Am 73:487–496. 10.1093/cid/ciaa653 10.1093/cid/ciaa653 PMC8326579 32756983

[4] Fu M, Hu L, Han K et al (2025) The burden of β-lactam allergy labels in health care: a systematic review and meta-analysis. Lancet Infect Dis. 10.1016/S1473-3099(25)00019-2 10.1016/S1473-3099(25)00019-2 40122092

[5] Wurpts G, Aberer W, Dickel H et al (2019) Guideline on diagnostic procedures for suspected hypersensitivity to beta-lactam antibiotics: guideline of the German Society for Allergology and Clinical Immunology (DGAKI) in collaboration with the German Society of Allergology (AeDA), German Society for Pediatric Allergology and Environmental Medicine (GPA), the German Contact Dermatitis Research Group (DKG), the Austrian Society for Allergology and Immunology (ÖGAI), and the Paul-Ehrlich Society for Chemotherapy (PEG). Allergo J Int 28:121–151. 10.1007/s40629-019-0100-8 10.5414/ALX02104E PMC7304290 32568254

[6] Brockow K, Wurpts G, Trautmann A et al (2023) Guideline for allergological diagnosis of drug hypersensitivity reactions. Allergol Sel 7:122–139. 10.5414/ALX02422E 10.5414/ALX02422E PMC10495942 37705676

[7] Brockow K, Pfützner W, Wedi B et al (2025) Recommendations on how to proceed in case of suspected allergy to penicillin/β-lactam antibiotics. Allergol Sel 9:28–39. 10.5414/ALX02531E 10.5414/ALX02531E PMC11905010 40083843

[8] Koch T, Leubner H, Brehm TT, Witte J (2023) Penicillinallergie: Sicher und effektiv ausschließen. Dtsch Ärztebl: A‑822–B-699

[9] Trubiano JA, Vogrin S, Chua KYL et al (2020) Development and validation of a penicillin allergy clinical decision rule. JAMA Intern Med 180:745–752. 10.1001/jamainternmed.2020.0403 32176248 10.1001/jamainternmed.2020.0403 PMC7076536

[10] Obuchowski NA (1998) Sample size calculations in studies of test accuracy. Stat Methods Med Res 7:371–392. 10.1177/096228029800700405 9871953 10.1177/096228029800700405

